# Effect of aerobic exercise and low carbohydrate diet on pre-diabetic non-alcoholic fatty liver disease in postmenopausal women and middle aged men – the role of gut microbiota composition: study protocol for the AELC randomized controlled trial

**DOI:** 10.1186/1471-2458-14-48

**Published:** 2014-01-17

**Authors:** Wu Yi Liu, Da Jiang Lu, Xia Ming Du, Jian Qin Sun, Jun Ge, Ren Wei Wang, Ru Wang, Jun Zou, Chang Xu, Jie Ren, Xin Fei Wen, Yang Liu, Shu Mei Cheng, Xiao Tan, Satu Pekkala, Eveliina Munukka, Petri Wiklund, Yan Qiu Chen, Qing Gu, Zheng Chang Xia, Jun Jun Liu, Wen Bin Liu, Xue Bo Chen, Yi Min Zhang, Rui Li, Ronald J H Borra, Jia Xin Yao, Pei Jie Chen, Sulin Cheng

**Affiliations:** 1 School of Kinesiology, Shanghai University of Sport, 200438 Shanghai, China; 2 Shanghai Shidong Hospital, Shanghai, China; 3 Clinical Nutrition Centre, Fudan University Huadong Hospital, Shanghai, China; 4 School of Physical Education and Coaching, Shanghai University of Sport, 200438 Shanghai, China; 5 Research Centre for Health Promotion, Department of Health Sciences, University of Jyväskylä, 40014 Jyväskylä, Finland; 6 Department of Health Sciences, University of Jyväskylä, 40014 Jyväskylä, Finland; 7 Yanji Health Care Service Centre, Yangpu District, Shanghai, China; 8 Wujiaochang Health Care Service Centre, Yangpu District, Shanghai, China; 9 Teaching Experiment Centre, Beijing Sport University, Beijing, China; 10 Department of Diabetes Control, Shanghai Municipal Center for Disease Control & Prevention, Shanghai, China; 11 Department of Radiology, Harvard Medical School, Massachusetts General Hospital, Boston, USA; 12 Department of Diagnostic Radiology, University of Turku and Turku University Hospital, Turku, Finland; 13 Department of Psychology, Tianjin University of Sport, Tianjin, China

**Keywords:** Liver fat content, Glucose metabolism, Lipid metabolism, Gut microbiota, Metabonomics, Human, Clinical setting

## Abstract

**Background:**

Pre-diabetes and non-alcoholic fatty liver disease (NAFLD) are associated with an unhealthy lifestyle and pose extremely high costs to the healthcare system. In this study, we aim to explore whether individualized aerobic exercise (AEx) and low carbohydrate diet (LCh) intervention affect hepatic fat content (HFC) in pre-diabetes via modification of gut microbiota composition and other post-interventional effects.

**Methods/design:**

A 6-month randomized intervention with 6-month follow-up is conducted from January 2013 to December 2015. The target sample size for intervention is 200 postmenopausal women and middle-aged men aged 50–65 year-old with pre-diabetes and NAFLD. The qualified subjects are randomized into 4 groups with 50 subjects in each group: 1 = AEx, 2 = LCh, 3 = AEx + LCh, and 4 = control. In addition, two age-matched reference groups (5 = pre-diabetes without NAFLD (n = 50) and 6 = Healthy without pre-diabetes or NAFLD (n = 50)) are included. The exercise program consists of progressive and variable aerobic exercise (intensity of 60 to 75% of initial fitness level, 3–5 times/week and 30–60 min/time). The diet program includes dietary consultation plus supplementation with a special lunch meal (40% of total energy intake/day) which aims to reduce the amount of carbohydrate consumption (30%). The control and reference groups are advised to maintain their habitual habits during the intervention. The primary outcome measures are HFC, serum metabolomics and gut microbiota composition. The secondary outcome measures include body composition and cytokines. In addition, socio-psychological aspects, social support, physical activity and diet will be performed by means of questionnaire and interview.

**Discussion:**

Specific individualized exercise and diet intervention in this study offers a more efficient approach for liver fat reduction and diabetes prevention via modification of gut microbiota composition. Besides, the study explores the importance of incorporating fitness assessment and exercise in the management of patients with pre-diabetes and fatty liver disorders. If our program is shown to be effective, it will open new strategies to combat these chronic diseases.

**Trial registration:**

Current Controlled Trials: ISRCTN42622771.

## Background

Non-alcoholic fatty liver disease (NAFLD) is becoming the most common cause of chronic liver disease in the developing world, and occurs in 17-30% of the population in Western countries and 2-4% worldwide [1]. In China the prevalence of NAFLD is 10-30% and up to 75% among obese and type 2 diabetes (T2D) [2]. At the same time, the prevalence of pre-diabetes has increased about 5-15% annually in different parts of China [3-5]. The main causes of pre-diabetes and NAFLD are related to lifestyle change due to modernization and both of them are largely preventable by changing the lifestyle.

It has been known for decades that gut microbes play an important role in metabolic, physiological and immunological processes in the human body [5-7]. Recently, studies have shown that unbalanced gut microbiota composition, i.e. dysbiosis, is associated with obesity-related metabolic disorders [8], T2D [9] and cardiovascular diseases (CVDs) [10] as well as longevity [11]. In addition, gut-derived bacterial components and metabolites are possible contributors to the onset of inflammation and further hepatic insulin resistance (IR) since the liver gains a substantial amount of nutrient- and metabolite-rich blood through the portal vein which links it to gut [12]. Recent evidence suggests a role for the gut microbiota both in the etiology and progression of hepatic fat accumulation (HFA), which is a multifactorial phenomenon associated with central obesity and metabolic syndrome [13,14].

The gut bacteria affect an individual’s ability to utilize energy from diet and when unbalanced microbiota composition can sustain a condition of subclinical chronic activation of the immune system, or so called systemic low-grade inflammation (SLGI), inducing insulin resistance and enhancing the metabolic risk [15]. On the other hand, in vivo studies show that some viruses such as enteroviruses can affect the metabolic status by directly infecting the liver and pancreas, while others such as adenoviruses can induce SLGI by infecting adipose tissue and interacting with mucosal immune system [16-18].

It is known that physical activity activates stomach and bowel function by altering local circulation and affects intestinal mucosal immunity. Interestingly, recent animal studies have shown that physical activity and diet cause compositional changes in gut microbiota [19,20]. Another study has shown that life-long calorie restriction of both high-fat and low-fat diet, but not voluntary exercise, significantly changed the overall structure of the gut microbiota of C57BL/6 J mice [11]. However, this study did not detail the intensity and duration of the exercise. Thus the effect of exercise on microbiota is still largely unknown. The mechanism underlying the effects of exercise and diet on gut microbiota needs to be established. Finally, data from human studies, though sparse, suggests that exercise can reduce liver fat [21] and thus the benefits of exercise may be mediated, in part, by a reduction in hepatic lipogenesis [22].

### Objectives and hypothesis

The gut microbiota composition can be modified by exercise and diet. Therefore, changes in the level of physical activity and composition of diet may result in favourable changes in microbiota composition which could, in turn, be accompanied by reductions in liver fat content, thus improving glucose and lipid metabolism and insulin sensitivity. A six-month exercise and diet intervention is enough to induce re-patterning of microbiota composition. However, “healthy” gut microbiota composition needs persistent exercise and balanced diet. Subjects may relapse to an “unhealthy” gut microbiota composition after cessation of exercise and balanced diet.

Therefore, the purpose of this study is to investigate the gut microbiota composition of postmenopausal women and middle aged men with pre-diabetic NAFLD compared to healthy controls, and whether chronic but latent bacterial infections are more frequent in NAFLD subjects than in healthy subjects. In addition, we will determine whether aerobic exercise and low carbohydrate diet reduce liver fat content via modification of gut microbiota composition in postmenopausal women and middle-aged men.

## Methods/design

### Trial design

**Figure 1:**
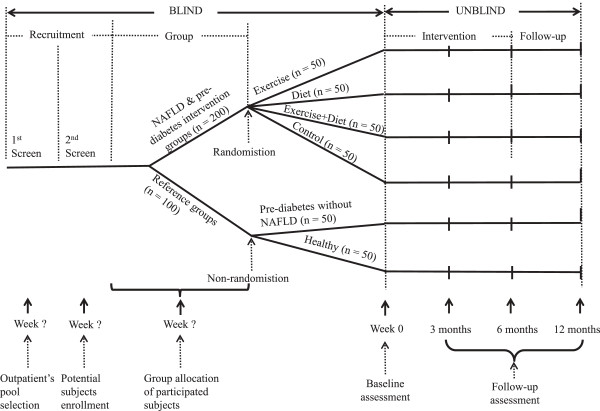
Design of the AELC-study.

This is a six-arm study with four-arm randomized controlled trial and an additional 2 arms as references (Figure 1). Participants are recruited from the outpatient registration pool of those who have participated in annual health checks for diabetes and liver fat during 2012 to 2013 at the Yangpu District Health Care Service Centres, Shanghai, China. After an initial screening, potential subjects will be assessed for inclusion in the run-in trial. Those subjects who meet the inclusion criteria will be then invited to the intervention and follow-up study for a total duration of 1 year (6-month intervention and 6 month follow-up). Those participants who are qualified for the intervention study will be randomized into 4 groups after baseline assessments. Those subjects who are qualified for the reference groups will not be randomized.

### Eligibility criteria

1. Inclusion criteria for intervention groups:

Men or women aged 50–65 years with fasting glucose between 5.6 to 6.9 mmol/or glucose between 7.8 to 11.0 mmol/L 2 hour after the intake of glucose (75 g), diagnosed as NAFLD by ^1^H MRS (liver fat >5%) [23] and by questionnaire that on-going or recent alcohol consumption is <21 drinks on average per week in men and < 14 drinks on average per week in women [24]; no chronic cardiovascular, serious musculoskeletal or gastrointestinal problems and not on extreme diets; and for women, serum follicle-stimulating hormone level greater than 30 IU/L and last menstruation more than 6 months ago but within 10 years.

2. Inclusion criteria for reference groups:

Reference group 1: Men or women aged 50–65years with fasting glucose between 5.6 to 6.9 mmol/L or glucose between 7.8 to 11.0 mmol/L 2 hours after intake of glucose (75 g), not be diagnosed to have NAFLD by ^1^H MRS (<5%), no chronic cardiovascular, serious musculoskeletal or gastrointestinal problems and not on extreme diets; and for women, serum follicle-stimulating hormone level greater than 30 IU/L and last menstruation more than 6 months ago but within 10 years.

Reference group 2: Men or women aged 50–65 years with fasting glucose below 5.6 mmol/L or glucose below 7.8 mmol/L after 2 hour in takes of glucose (75 g), no NAFLD by ^1^H MRS (<5%), no chronic cardiovascular, serious musculoskeletal or gastrointestinal problems and not on extreme diets; and for women, serum follicle-stimulating hormone level greater than 30 IU/L and last menstruation more than 6 months ago but within 10 years.

3. Exclusion criteria:

Body mass index (BMI) > 38 kg/m2; serious cardiovascular or musculoskeletal problems; diagnosed Type 1 diabetes and T2D; and mental illness.

### Study settings

The laboratory tests and interventions are performed and managed at the Department of Sport Medicine, Shanghai University of Sport, the Yangpu District Health Care Service Centres and the Department of Endocrinology, Shidong Hospital, and Clinical Nutrition Centre at Fudan University Huadong Hospital, Shanghai, China.

### Interventions

1. Exercise group:

Specific supervised individualized exercise (mainly aerobic exercise such as Nordic brisk walking + stretching and other group exercise) programs will be developed by an exercise researcher after baseline assessments on the basis of each individual’s fitness level. The supervised exercise program will be progressive and variable and will be monitored by an exercise researcher during the course of the study. The intensity and duration of exercise will be increased from 60% to 75% of the maximum oxygen uptake (estimated from fitness test) and from 30 to 60 min per session, and the frequency from 3 to 5 times a week, and will be updated monthly.

2. Diet group:

Dietary programmes are developed after baseline assessments on the basis of each individual’s current dietary intakes and body weight. Overweight/obese participants are advised to moderately reduce their total energy intake (by 300–500 kcal per day for the first 3 months) with guidance on the proportion of macronutrients to be consumed. The target is to reduce body weight by 3 kg in the first 3 months of the intervention. After this period, the participants are advised to maintain their achieved body weight reduction, and continue to gradually reduce their body weight towards normal levels, with a target of a 10% reduction from their initial body weight. Participants with normal weight are advised to maintain their body weight.

During the intervention, each meal is planned by a clinical nutritionist. We provide lunch daily to each participant during the course of the intervention. The lunch accounts for 40% of the total daily energy intake. The breakfast and dinner each account for 30% of the total daily energy intake. The proportion of macronutrients are planned as 30-40% carbohydrate with < 5% sucrose and with 18-20% as fiber, 40% fat (SAFA 10%, MUFA 15-20%, PUFA 10%) and 20% protein. The lunch will be prepared under the guidance of a clinical nutritionist at the Student Restaurant of Shanghai University of Sport. Each meal will contain three to four dishes of foods commonly eaten by Chinese families in Shanghai. A trained study staff member will weigh each item for each specific person according to the dietary plan and the cooked dishes will be put into a named lunch box for each participant. The lunch box will be then delivered to the study district office where the study subjects are gathered and the eating will be monitored. If the participants don’t have time to come to the lunch, the lunch box will be delivered to their home and we will ask the participants to take it as their dinner.

The subjects will cook their breakfast and dinner by themselves and eat the proportion following the nutritionist advices. They will take the pictures of each meal by an iPhone provided by the research. A researcher will upload the pictures weekly to our data management server for further evaluation.

3. Exercise + diet group:

The AEx + LCh group will perform the same exercise program and follow the same diet program as abovementioned for AEx and LCh groups.

4. Control and reference groups:

The control and reference groups will be advised to maintain their level of physical activity and habitual eating during the intervention. After intervention, we will provide to the control group an opportunity to participate in our planned exercise and diet intervention for 3 months.

### Primary and secondary outcomes

#### Primary outcomes

1. Liver fat content

2. Serum metabolomics, level of glucose, insulin, triglycerides and free fatty acids

3. Gut microbiota composition

The measurements of liver fat content, variables related to glucose metabolism, triglycerides and free fatty acids will be assessed at baseline and 6-month follow-up; gut microbiota composition will be measured at baseline, 3-month and 6-month time points.

### Secondary outcomes

1. Questionnaires of behavioural characteristics, diet, physical activity, health condition and medications

2. Anthropometry (height, body weight, body size demission of different region of body)

3. Blood pressure

4. Body composition (fat mass, lean mass and bone mass)

5. Physical fitness and heart rate (2 km walk and 3 minutes step tests)

6. Muscle strength (maximal isometric voluntary contraction of the right grip, left elbow flexors and left knee extensors)

7. Venous blood sample (total cholesterol, HDL-cholesterol, LDL-cholesterol, apolipoproteins A-I and B, lipoprotein(a), cholesterylester fatty acid composition, hormones, cytokines and serum metabonomics)

The secondary outcome measures will be assessed at baseline, 3- and 6-month time points except for body composition (fat mass, lean mass and bone mass) and blood sample which will be performed at the baseline and 6-month time points.

### Measurements procedures

1. Screening:

The purpose of the screening is to verify the eligibility of potential participants. The recruitment of participants will be done at Yangpu District Health Care Service Centres and the Department of Endocrinology, Shanghai Shidong Hospital Shanghai, China. The potential subjects will be selected from the outpatient pool of those who have records of ultrasound for evaluation of liver fat and fasting blood results for diagnosing diabetes from 2012 to 2013. Registered nurses from health care centers will first contact the potential subjects and invite them to a study information meeting. Detailed information of the objectives of the study, its nature and constraints, the anticipated risks and the expected benefits are given by the investigating physician of endocrinology and professor of sport medicine in the recruitment information meetings. When the screening consent form has been signed, patients will be asked to fill in a screening questionnaire to check their health and medication background as well as alcohol consumption, and to undergo a glucose tolerance test (blood draw after overnight fasting, followed by samples of 30 minutes and 2 hours after the intake of 75 g of glucose).

2. First visit of the Run-in Trial:

The run-in trial consists of an 8-week period during which the participants who are qualified in terms of the screening questionnaire will complete all of the forms and protocols required for successful entry to the 1-year study. The purpose of the run-in trial is to identify and exclude those individuals who cannot or will not fulfil the requirements of the study. This should decrease the dropout rate of the 1-year study. We anticipate that approximately 50% of potential participants will not complete the run-in trial with sufficient detail or completeness to be entered in the study.

In the first visit of the Run-in Trial, those subjects qualified by screening (subjects who have fasting glucose level between 5.6 and 6.9 mmol/L or after 2-h intake of glucose between 7.8 to 11.0 mmol/L are willing to participate in this study will be invited for further blood sample collection. In addition, the pre-diabetes and healthy reference groups will undergo the same procedure. During the first visit, the participants will receive an in-depth explanation of the 1-year study and informed consent for the intervention and follow-up study will be obtained in writing.

3. Second visit of Run-in Trial:

At the second visit, a physician and a researcher will examine all the results of the first visit to verify that the subject meets the inclusion criteria. If a person is qualified for participation in the study, he or she will be invited to the laboratory at the Department of Sport Medicine, Shanghai University of Sport for demographic, health history and physical activity questionnaires, height, weight, waist circumference, blood pressure, physical performance, body composition by DXA, as well as instructions for fecal sample and 24 hours urine collections. They will be also invited to have a magnetic resonance spectroscopy (^1^H MRS) scan to evaluate whether they have NAFLD (>5% liver fat content). The qualification of the participants will be established after this visit and randomization will be performed.

4. Randomization visit:

When the qualified subjects are randomized, an information meeting will be organized for each group. Detail of the activities during intervention will be explained by a principle investigator or a co-principle investigator. For the AEx group, we will provide walking poles and teach them how to use it. A heart rate monitor (M5 Suunto Oy, Finland) will be provided during each exercise session to monitory the intensity and duration of exercise. For the LCh group, a dietary consultation will be organized to instruct subjects how to prepare their breakfast and dinner and what we are going to provide them for lunch during the intervention. In addition, we will provide a mobile phone to each subject and teach them how to take pictures of their breakfast and dinner. For the AEx + LCh group, we will provide instructions as abovementioned both for exercise and diet groups. For the control group and both reference groups, we will emphasize the importance of their participation in study and instruct them to maintain their habits during the intervention. All groups will be instructed to fill a daily diary regarding their daily activities and a step counter will be provided to them to monitory their daily walking status.

5. Follow-up visits:

The purpose of the follow-up visits is to monitor the compliance, enhance retention of the subject in the study, and obtain information on factors that may affect outcomes of the study. After the randomization visit, a study coordinator will contact participants every month to check their exercise records and update the exercise program. The study coordinator will also give feedback of their dietary intake on the basis of the images of meals and snacks provided by the subjects themselves. The participants will have follow-up visits to the laboratory of Shanghai Sport University at months 3, 6, 12. Their height, weight, food frequency questionnaire, physical activity record, fitness tests, blood pressure, blood samples, fecal samples, urine samples and changes in health status will be checked at those time points. MRI and DXA will be included in the follow-up visits for months 6 and 12.

### Methods of measurements

#### Questionnaire, anthropometry and physical examinations

Background information regarding lifestyle, behavioural and motivational characteristics as well as medical history will be collected by questionnaires. Data gathered from eligible subjects will then be used to describe the study populations, and individual results will be used to screen the changes and for monthly feedback.

Daily physical activity will be recorded through an activity diary (designed by the study) during the 6-month intervention and 6-month after intervention. In addition, food records will be collected 12 times to estimate the subject’s energy intakes and intake of different nutrients during the study for all groups. The collected information will be evaluated by exercise and nutrition experts, and feedback will be given by each individual’s family doctor.

Height will be determined using a wall-fixed measuring device, and weight using a calibrated scale. Height and weight will also be used to determine body mass index (BMI, weight(kg)/(height(m))^2^. Blood pressure will be measured after 5 min rest. A physician will examine the physical conditions of subjects and ensure that subjects meet the inclusion criteria.

#### Blood samples

Glucose tolerance tests will be performed after overnight fasting, 30 minutes and 2 hours after the intake of 75 g glucose for the assessment of serum insulin (chemiluminesent immunoassay) and glucose (automatic biochemistry analyzer).

Venous blood samples will be taken in standardized fasting conditions at 7–8 a.m. Total cholesterol, high-density lipoprotein (HDL), low-density lipoprotein (LDL), triglycerides, apolipoproteins A-I and B, lipoprotein (a), glucose, insulin, cholesterylester fatty acid composition, free fatty acid profile and sex hormones will be measured from serum sample by conventional methods. We will use appropriate methods to measure a range of factors related to inflammation. ELISA or Western Blotting can be used to assess serum samples for leptin, adiponectin, resistin, tumor necrosis factor alpha (TNFα), C-reactive protein (CRP), interleukin (IL-6), interleukin 1β (IL-1β), interleukin-1 receptor antagonist (IL-1Ra), and angiotensinogen, the fibroblast growth factor 23 (FGF23), Zn-metalloendopeptidase (PHEX), matrix extracellular phosphoglycoprotein (MEPE), and cartilage oligomeric matrix protein (COMP). Plasma samples will be used to assess lipopolysaccharides (LPS) concentration by Endosafe-MCS (Charles River laboratories, Lyon, France) based on the Limulus amaebocyte Lysate (LAL) kinetic chromogenic methodology that measures color intensity directly related to the endotoxin concentration in a sample.

Serum free fatty acid profile measurement will be performed in order to define the different fractions and ratio of saturated, mono-unsaturated and polyunsaturated fatty acids. The obtained information is needed to estimate certain de novo lipogenic enzymes activity by comparing the ratio of different precursors and end-products fatty acid metabolism in the serum. The information is also needed to evaluate how diet, physical exercise and liver fat affect the concentrations of free fatty acids and their ratios in the serum. Gas chromatography-mass spectrometer will be used for the analysis.

#### Fecal samples collection

The fecal samples (two samples per each time) will be collected during the second day of the 3-day dietary records collection. Detailed sample collection and transportation instructions are given by a study nurse. The study will provide to subjects the materials for sample collection and transportation from home to health care centre which including: 1 cool box, 2 plastic specimen jars (the cover of the jar contains the spatula), plastic bag, 4 cold chargers and plastic gloves. The subjects are advised to sample approximately 2–3 grams of feces by spatula attached to the cover of the plastic specimen jar. Then put fecal samples into the specimen jar and close it. The jars are stored in a plastic bag with the cool box. After samples are collected, the subjects will immediately deliver the samples to the health care centre (mostly about 5–10 min walking distance). If the subjects are not able to deliver the sample by themselves, the will call our research line and a personal from research group will pick up the samples from subjects home within 30 min to 1 hour.

Fecal samples are frozen after collection, stored at −80°C, and the DNA will be extracted with a fully automated instrument using magnetic beads-based technology (Genoextract, Hain Lifescience GmbH, Germany). Whole-genome sequencing will be performed using Illumina Hiseq 2000 system. In addition, fecal bacteria composition will be analyzed with a method based on flow cytometry, 16S rRNA hybridization, and DNA-staining. Different bacterial indices will be calculated to evaluate the inter-relationship of different bacterial species and how they are associated with liver fat content [8]. Concentrations of short chain fatty acids (SCFAs) will be determined in the supernatant using an Agilent 6890 N gas chromatograph with flame ionization detection and using ChemStation software for data processing.

#### Urine sample collection

Twenty-four hours urine samples will be collected in the morning after avoiding the first sample during the 2^nd^ to 3^rd^ day of the 3-days dietary records collection. The detailed instruction on urine sample collection will be given to the subject by a study nurse. Special sample collection device will be provided to the subjects. The device allows us to store small proportion of the total sample for whole day including information of total volume of samples. The sample will be stored at −80°C until analysis. The function of kidney and the concentration of minerals will be analysed.

#### Abdominal fat assessment

For abdominal fat assessment, axial T1-weighted dual-echo (in-phase and out-of-phase) Volumetric Interpolated Breath-hold Examination (VIBE) images will be obtained of the entire abdominal area with a single breath-hold using the following parameters: repetition time (TR) 4.36 ms, first echo time (TE1) 1.33 ms (out-of-phase) and second echo time (TE2) 2.45 ms (in-phase), slice thickness 4.8 mm, voxel size 1.9×1.3×4.8 mm, distance factor 20%, matrix 320 × 208, field of view (FoV) 40 cm, flip angle (FA) 9 degrees and average acquisition time of 15 s. Areas of abdominal subcutaneous, visceral and retroperitoneal fat will be semi-automatically segmented using the OsiriX software package (OsiriX Foundation, Geneva, Switzerland). Measured volumes of each of the abdominal adipose tissue compartments will be converted into tissue mass taking into account slice thickness and an adipose tissue density of 0.9196 g/ml [25].

#### Hepatic fat content assessment

Non-invasive measurement of hepatic fat content (HFC) will be performed using single-voxel proton magnetic resonance spectroscopy (^1^H MRS). The ^1^H MRS measurements will be performed on a 3 T Siemens Magnetom Verio scanner (Siemens Medical Solutions, Erlangen, Germany) using the appropriate elements of the Body Matrix and built-in spine coil as the receiving elements. A respiratory (bellows) gated point-resolved spectroscopy (PRESS) sequence with the following parameters will be used: TR 3000 ms, TE 30.0 ms, number of averages 24 (for both suppressed and unsuppressed water signals) and typical acquisition time of 1 min 24 s. Obtained spectra will be analysed using the Linear Combination of Model spectra software suite which is generally considered to be the gold standard for in-vivo spectroscopy analysis [26]. The fat and water spectrum signals will be corrected for differences in T2 decay, molar concentrations of ^1^H nuclei in fat and water and HFC will be defined as fat in relation to the total weigh of liver tissue, as previously described [23].

#### Body composition

Dual-energy X-ray absorptiometry (DXA Prodigy, GE Lunar Corp., Madison, WI USA with software version 13.60.033) will be used to estimate lean tissue mass (LM), fat mass (FM) and bone mass (BM) of the whole body, legs, arms, and trunk as well as android and gynoid regions. All metal items will be removed from the participants to ensure the accuracy of the measurement.

#### Fitness test and heart rate

2 km walking test will be performed by walking 2 kilometres as fast as possible on a flat surface [27]. The result is recorded as a fitness index taking into account person’s age, gender, height, weight, time taken to walk 2 kilometres and the heart rate at the end of the test. There are five fitness classes which can be used to compare result with the fitness of others of the same age and gender or a person’s own previous results and development between the tests. The individual’s result will be used to set up their own personalized exercise program.

The YMCA Step Test is a relatively low stress effort and therefore can be used for unfit individuals [28]. Beat-by-beat heart rate will be recorded continuously during 3 minutes exercise, and in a sitting posture at rest before exercise, and 1 minute after exercise [28].

#### Muscle strength test

Maximal isometric voluntary contraction (MVC) of the right hand grip strength, left elbow flexors and left leg extensors will be measured in a sitting position with an adjustable dynamometer chair (Good Strength). Subjects will be encouraged to exert their maximal effort during an isometric test for ~3 s. The MVC of the hand grip strength, elbow flexors will be measured with the subject’s forearm strapped at the wrist using Velcro straps with elbow at 90° and the thumb in an upward position. Seat height will be adjusted to ensure that the upper arm is at a 90° to the chest and the elbow is at the pivot point of the apparatus. In the measurement of MVC of knee extensors, the subject’s lower leg will be strapped at the distal end of the lower leg using Velcro straps with knee at 120°. Seat height will be adjusted to ensure that the hip will be at 90° to the trunk and the knee will be at the pivot point of the apparatus. The hip will be also strapped by Velcro straps to limit its movement.

#### Concomitant adherences and procedures

During the intervention, an exercise instructor will ensure that each individual has the right starting level and will follow their progress during the entire trial. We will organize the participants into groups according to their time and places of residence. Exercising together as a team will increase their compliance. A group leader of each 10 subjects will be selected by participants to assist the instructor to ensure a high participation rate. The exercise instructor will be with the subjects at least three times a week during the intervention. Group feedback meetings will be arranged every 3 months during the trial by an exercise specialist.

To ensure good compliance in diet intervention, we provide a mobile phone to the subjects to allow them to take photographs of their breakfast and dinner daily during the study. The photos are downloaded by a researcher weekly. The study nutritionist analyses dietary composition from the uploaded photos and gives feedback to each participant monthly. In addition, food diary information is also collected at the 3-month time point in order to give more dietary suggestions to the subjects. A clinical nutritionist will hold nutritional education meetings three times for the diet and AEx + LCh groups during the intervention period.

For the control and reference groups, we will keep in contact with them via phone call monthly and questionnaire to check on their physical condition and lifestyle at 3 and 6 months follow-up during the intervention.

#### Sample size estimation

The sample size calculation (drawing on previous literature) with an estimate of how many participants will be needed for the primary outcome to be statistically, clinically and/or politically significant. For the specific primary outcome of liver fat and microbiota, based on our plot study, 34 subjects in each group would have 85% power for mean comparison between the randomized groups for the liver fat. We set the significance level at 0.05 and allowed for 10 mean comparisons with the Bonferroni correction between the groups. Taking into account multi-variable comparisons in the analysis, we increase our simple size to 50 in each group. We also taking into account the compliance and drop-out (15%), thus the total number of subjects was estimated to meet each specific aim.

In addition, typical projection-based methods used to analyze whole-genome data, such as Principal Component or Partial Least Squares Regression, do not have statistical inference methods. Thus, the corresponding methods for sample size estimation are data-driven methods involving latent variables. However, the large dimension and the time series nature of our datasets provide a good opportunity for inferring associations between different measurements. By using a systems biological approach we can develop models based on path analysis, which comprehensively integrate data for analysis. With such models we will be able to reveal the relationships between different variables.

### Randomization methods

1. Randomization Process:

A computer program will be used for generating the randomization assignment for this study by a statistician. 10 blocks of 20 randomization numbers will be generated and sealed in an envelope and kept by the study coordinator. On the basis of subjects’ enrolment time when 20 qualified subjects are reached, the study coordinator will open one block to allocate subjects into one of the 4 groups (target 50 persons per group, see Figure 1):

2. Blinding:

1) Exercise group (AEx)

2) Diet group (LCh)

3) Exercise + Diet group (AEx + LCh)

4) Control group (Con)

In addition two references groups are not in randomization:

5) Pre-diabetes without NAFLD (Ref1)

6) Healthy reference group (Ref2)

Since lifestyle intervention is not possible for subjects to be blinded, we have designed the study in such way that the tests and analyses will be blinded for investigators during the study.

The study coordinator will be blinded from which group the participants are signed in. The accuracy of the randomization log is the responsibility of the study coordinator.

Investigators will be blinded with respect to the randomization assignment of each participant. The investigators will remain blinded until the completion of the study.

### Statistical methods

All data collected by questionnaires will be entered into database management software (Filemaker Pro version 10, FileMaker Inc. Santa Clara, CA, USA), double checked by independent researchers, and exported to PASW statistics version 20 (IBM Corporation, USA). If data is not normally distributed, their natural logarithms will be used for further analysis. Descriptive statistics will be used to present the background and anthropometric data at the baseline and follow-up assessments as mean and 95% confidence interval (CI) unless otherwise stated.

An intention-to-treat (ITT) analysis will be performed to compare the AEx, LCh and AEx + LCh groups to Con group. The effects of the interventions will be assessed using analysis of variance (ANCOVA) for repeated measures (treatment group × time) and baseline difference as a covariate. If the significance of the group by time interaction is p < 0.05, the effect will be localized utilizing Bonferroni for multiple comparisons. The level of statistical significance chosen for the contrasts will be p < 0.05. In addition to the ITT analysis, efficacy or active treatment analysis will be done when the compliance of the participation of the intervention is ≥60% of the whole trial and for <60% not more than 3 months continuously [ANCOVA for repeated measures (treatment group × time) and baseline difference as a covariate.] The percentage differences (0–6month) will be calculated from duration between baseline and end point measurements for each individual. The comparison of percentage changes in different groups will be performed using ANCOVA (two factor interactions: compliance/noncompliance × treatment group) controlled for the baseline value using Bonferroni for multiple comparisons. If the significance of the overall group difference is p < 0.05, then the effect will be localized by contrast to the Con group. When the 95% CI does not include zero, the difference is regarded as statistically significant at α = 0.05.

Linear Pearson’s correlation, partial correlation, Kendall Tau’s, and bivariate, logistic, and multivariate regression analysis will be used for analysing relationships. In addition, systems biology approaches will be used to develop models which integrate the different types of data and the high-throughput data measured in terms of gut microbiota composition and fecal SCFAs content, as well as LPSs and inflammation.

### Ethical issues, research permits or information permit applications

The current study will continue to adhere to all relevant guidelines for good scientific and clinical practice. All subjects in this study are volunteers. None of the measurements are known to have any significant health risk. The benefits and associated risks of the study will be carefully explained to the subjects and voluntary participation will be stressed. Informed consent will be obtained from all subjects. The study physician will be available during the laboratory tests. The study has been approved by Ethics Committee of Shanghai Institute of Nutrition, 06.01.2013, ref: 2013–003 and has registered in International Standard Randomized Controlled Trial Number Register (ISRCTN 42622771).

All data will be handled and archived confidentially. All the samples will be coded by ID number without personal information. All image scans and background information will be electronically transferred and stored in the specific database. The blood samples and fecal samples will be stored at -80°C. If the analyses will be performed outside the data collection site, the sample transportation will be handled according to the relevant safety standards. The duration of the sample storage is generally 10 years. If the samples storage time are exceeds the permitted time, a new permit will be obtained from both the subjects and the Ethical committee. If the subjects are deceased, permission will be sought from the relatives.

## Discussion

NAFLD is a very prevalent and severe disease which can lead to cirrhosis, liver carcinoma and death related to liver morbidity [29]. Meanwhile, epidemiological studies showed that 25% of patients with pre-diabetes progress to type 2 diabetes in 5 years [30] and several prospective studies even suggested that the rate may be even higher, averaging 10-12% annually [31-33]. Recent studies have reported novel mechanisms for the pathogenesis of NAFLD and diabetes, especially involving the gut microbiota [34,35]. However, the studies that have suggested a role of the gut microbiota in NAFLD and diabetes have been mainly conducted in animal models [35,36]. In addition, it is not clear whether NAFLD is due to impaired glucose metabolism or vice versa. There are very few studies examining the link between gut microbiota and the development of NAFLD in humans. Moreover, the relationship between gut microbiota composition and different nutritional status and physical activity has been explored mainly in animal models, but not in humans [36]. To the best of our knowledge, the AELC-study is the first study that includes specific lifestyle intervention attempting to modify patients’ diet and physical activity, which are expected to change the gut microbiota composition and therefore reduce the severity of NAFLD and prevent diabetes.

We expect that both exercise and diet intervention will induce favourable changes in gut microbiota composition thus improving metabolic state, and further reducing liver fat content and prevent development of diabetes. We also anticipate that the combined exercise and diet intervention will be more effective intervention than exercise or diet alone. The further follow-up after intervention of the study will serve as a pilot for future large scale studies to evaluate how, in practice, exercise and diet programmes can sustain their positive impacts on health in these high risk populations.

From a clinical point of view, using lifestyle intervention offers a novel approach for liver fat reduction and diabetes prevention through modification of gut microbiota composition, and highlights the importance of incorporating fitness assessment and prescription in the management of patients with fatty liver disorders [21,22,37] and high risk of diabetes [9]. However, the challenge is how to motivate the inactive people who are at high risk for metabolic disorders and who really need to escape from unhealthy lifestyles, to start and, more importantly, to maintain regular exercise. Therefore, this study will focus also on implementing strategies to promote behaviour change including regular contact and assessment with a health care professional, self-monitoring, and personalization of goals to change physical activity and unhealthy diet behaviour.

This research using specific lifestyle interventions offers a novel approach for liver fat reduction and diabetes intervention via modification of the gut microbiota composition, and explores the importance of incorporating fitness assessment and exercise in the management of patients with pre-diabetes and fatty liver disorders. If our program is shown to be effective, it will open new strategies to combat these chronic diseases.

## Trial status

Participant recruitment started in January 2013. Baseline measurement started in March 2013 for the first enrolled group. Exercise, Diet, and Exercise plus diet interventions started in March 2013. The recruitment of subjects is continuing until June 2014.

## Abbreviations

1H MRS: Proton magnetic resonance spectroscopy; AEx: Aerobic exercise; BM: Bone mass; BMI: Body mass index; COMP: Cartilage oligomeric matrix protein; Con: Control group; CRP: C-reactive protein; CVDs: Cardiovascular diseases; DXA: Dual-energy X-ray absorptiometry; FM: Fat mass; FGF23: Fibroblast growth factor 23; HDL: High-density lipoprotein; HFA: Hepatic fat accumulation; IL-1Ra: Interleukin-1 receptor antagonist; IL-1β: Interleukin 1β; IL-6: Interleukin; IR: Insulin resistance; LCh: Low carbohydrate diet; LDL: Low-density lipoprotein; LM: Lean tissue mass; LPS: Lipopolysaccharides; MEPE: Matrix extracellular phosphoglycoprotein; MRI: Magnetic resonance image; MVC: Maximal isometric voluntary contraction; MUFA: Monounsaturated fattyacids; NAFLD: Non-alcoholic fatty liver disease; PHEX: Zn-metalloendopeptidase; PUFA: Polyunsaturated fatty acids; Ref1: Pre-diabetes without NAFLD group; Ref2: Healthy reference group; SAFA: Saturated fatty acids; SCFAs: Short chain fatty acids; SLGI: Systemic low-grade inflammation; T2D: Type 2 diabetes; TNFα: Tumor necrosis factor alpha.

## Competing interests

The authors’ declare that they have no competing interests.

## Authors’ contributions

SC is the principal investigator (PI). She designed the study and will oversee the project implementation, train researchers and doctoral students, participating in data collection, analyses and interpretation and writing publications. PJC, DJL, WYL, JG and JQS are the co-PI, they participated in study design and will implement the designed protocol, participating in supervise students, prepare the exercise and dietary programs, data collection, data analyses and interpretation and writing publications. JG, XMD, QG, ZCX, JJL, WBL, XBC, RL, RJHB are the study physician, they are responsible for physical examinations and patient counselling, recruitment and blood sample collections and MRI examinations, data analyses and interpretation and writing publications. JQS, YQC are responsible for dietary intervention plan. RWW, RW, JZ, CX, JR, XFW, YL, SMC, XT, SP, EM, PW, YMZ and JXY will participate in questionnaire, body composition, fitness tests, biological sample collection, exercise and diet intervention supervising and monitoring, data analyses and interpretation and writing publications. RL is responsible for quality control of the data collection and oversees the project implementation follow the good clinical practise guidelines. All authors read and approved the final manuscript.

## Pre-publication history

The pre-publication history for this paper can be accessed here:

http://www.biomedcentral.com/1471-2458/14/48/prepub
